# Supplementary material for: Sustainable environmental remediation via biomimetic multifunctional lignocellulosic nano-framework
Source: Nat Commun. 2022 Jul 28;13:4368. doi: 10.1038/s41467-022-31881-5 (PMC9334262; doi:10.1038/s41467-022-31881-5)
Supplement: Supplementary file 1 — Supplementary information [file 41467_2022_31881_MOESM1_ESM.pdf]

Supplementary information

Sustainable Environmental Remediation via Biomimetic Multifunctional Lignocellulosic Nanoframework Synergizing Effective Biodegradation

Jinghao Li[1,2,3], Xiaohan Li[1,2], Yabin Da[4], Jiali Yu[1,2], Bin Long[1,2], Peng Zhang[1,2], Christopher Bakker[3], Bruce A. McCarl[4], Joshua S. Yuan[5], and Susie Y. Dai[1,2*]

[1]Synthetic and Systems Biology Innovation Hub, Texas A&M University, College Station, TX 77843, USA

[2]Department of Plant Pathology and Microbiology, Texas A&M University, College Station, TX 77843, USA

[3]Department of Chemical Engineering, Texas A&M University, College Station, TX 77843, USA

[4]Department of Agricultural Economics, Texas A&M University, College Station, TX 77843, USA

[5]Department of Energy, Environmental, and Chemical Engineering, Washington University in St. Louis, St. Louis, MO 63130, USA

*For correspondence: sydai@tamu.edu

Supplementary note 1: PFAS adsorption kinetics. The adsorption kinetic study was determined by an individual batch experiment using 50 mL polypropylene bottles at a pH value of 7.0 ± 0.5. The 100 mg/L PFOA and PFOS solution were prepared for the adsorption kinetics test with 25 mg/L cellulose fiber, cellulose nanofibrils, modified lignin, cellulose fiber/lignin, cellulose fiber/modified lignin, cellulose nanofibrils/lignin, and RAPIMER composites, respectively. The mixtures were shaken in an orbit shaker at 150 rpm at room temperature (23 ±1 °C) before the adsorption test. After 20 mins stabilization, all samples were collected using 0.2 μm polypropylene syringe filter from supernatant at 0h, 0.5h, 1h, 2h, 4h, 4h, 8h, 8h, 16h, 16h, 32h, and 32h for the PFAS concentration test by High-Performance Liquid Chromatography Mass Spectrometry (HPLC-MS) assay, respectively. Experiments were performed in triplicates, due to the small variations, all points were reported instead of the standard deviation. For the adsorption kinetics model, the pseudo-first-order model (eq. 1) was used to fit the experimental results as follows:

$$\frac{t}{q_t} = \frac{1}{kq_e^2} + \frac{t}{q_e} \quad (1)$$

where q_t (mg/g) is the quantity of PFOA and PFOS adsorbed at different contact time t, respectively. q_e (mg/g) is the equilibrium adsorption capacity in different batch, and k is the pseudo-second-order rate constant (mg/g/h).

Supplementary note 2: PFAS adsorption isotherms. The concentration of RAPIMER and modified lignin sorbent were fixed at 20 mg/L, and the initial PFOA and PFOS concentrations were 25, 50, 75, 100, 150, 200, 300, 400 mg/L, respectively. The batch isotherm experiments were performed in 50 mL polypropylene (PP) bottles, and the mixtures were shaken at room temperature (23 ±1 °C) in an orbit shaker at 150 rpm for 24h to reach equilibrium. After 20 mins stabilization, samples were collected using 0.2μm polypropylene syringe filters from the supernatant and were transferred to LC vials for LC-MS. Control experiments were performed in the same condition with no addition of sorbent, and no losses were observed. Experiments were performed in triplicate, due to the small variations, all points were reported. The

Langmuir model (eq. 2) and Freundlich model (eq.3) were employed to fit the adsorption reaction of PFOA and PFOS on RAPIMER and modified lignin sorbent, respectively.

$$q_e = \frac{q_m b C_e}{1+bC_e} \quad (2)$$

$$q_e = K_f C_e^{1/n} \quad (3)$$

where q_e (mg/g) is the adsorption capacity, C_e (mg/L) is the equilibrium concentration, q_m (mg/g) is the maximum capacity of adsorbate required to form a complete monolayer on the surface and b is the Langmuir constant. C_e/q_e was plotted against C_e and the data was fit to linear regression model, q_m and b constants can be calculated from the slope and intercept. The Freundlich constant K_f is related to the adsorption capacity of the materials, and $1/n$ is a constant related to surface heterogeneity. When log q_e is plotted against log C_e and the data analyzed by linear regression, $1/n$ and K_f constants were determined from the slope and intercept.

Supplementary note 3: Effect of PH. The effect of pH on PFOA and PFOS adsorption was examined on the RAPIMER and modified lignin, respectively. The experiments were run at pH values of 4, 6, 8, and 10 by adding specific amounts of 0.1 M NaOH or HCl solutions. The RAPIMER or modified lignin (25 mg/L) and PFOA or PFOS solution (100 mg/L) were added to 20 mL DI water with different initial pH values in 50 mL polypropylene bottles. The bottles were shaken at room temperature (23 ± 1 °C) on an orbit shaker at 150 rpm for 48h. Blanks without RAPIMER nor modified lignin were run as controls. The samples were then collected from the supernatant, filtered with 0.2 µm polypropylene filters, and then transferred to HPLC-MS for further measurement. All samples were run in triplicates.

The pH point of zero charge (pH_{pzc}) was measured as follow. The RAPIMER (25 mg) or modified lignin sorbent (25 mg) were added to 5 mL 0.1 M NaCl solutions with different initial pH values of 2, 5, 8 and 11. DI water was boiled to remove dissolved CO_2 to prepare the solutions. The samples were shaken for 48h at 150 rpm at room temperature to reach equilibrium. After the materials were completely settled, the final pH of the supernatant was measured as pH_{pzc} of the material. The control of the different pH solutions

without sorbents were performed for comparison and no change of pH value was observed. Experiments were performed in triplicates. Due to the small variations, the points were overlapped and showed in the figure.

Supplementary note 4: PFOA and PFOS adsorption in flow conditions and in the presence of co-contaminants. The RAPIMER and modified lignin were set up to test the low concentrated PFOA and PFOS adsorption, respectively. PFOA and PFOS solutions (20 mL) at 1 µg/L and 10 µg /L concentrations were used in a flow setting. The RAPIMER (10 mg) and modified lignin sorbents (10 mg) were separately weighed and packed into customized polypropylene syringe filters. The filters were installed on a polypropylene syringe (Supplementary Figure 8). The PFOA and PFOS solutions were pressurized via a pump to go through the syringe at a 1 mL/min flow rate. The effluent solutions were collected, filtered with 0.2µm polypropylene filters, and then transferred to HPLC-MS for quantitative measurement. For the PFAS adsorption in the presence of co-contaminants, natural rainwater was collected in a large plastic cooler at College Station, Texas. After removing foreign objects such as leaves, insects, and large particles, the rainwater was filtered using a 0.22 µm membrane filter. Anionic dye (direct red 81), chromium (Cr), cadmium (Cd), copper (Cu), lead (Pb), and PFOA/PFOS (50:50) solutions were spiked into the rainwater to reach a final concentration of one µg/mL for each individual contaminant. The same flow setting was used for contaminant adsorption testing (Supplementary Figure 8). The influent and effluent were collected for the contaminant adsorption tests using HPLC-MS (PFOA and PFOS), optical design (OD) measurement (510nm for dye), and ICP-MS (Cr, Cd, Cu, and Pb). The results were reported in Fig. 3f.

Supplementary note 5: Quantitative PFAS analysis by high-pressure liquid chromatography-mass spectrometry (LCMS). Filtered solutions or PFOS and PFOA standard solutions (10µL) were loaded into a 3.0 mm × 50 mm (1.7 µm) Acquity UPLC BEH C18 column (Waters, MA, USA) to separate the compounds. An ammonium acetate aqueous solution (20 mM, solvent A) and 100% Methanol (solvent

B) were used as mobile phases, with a flow rate of 300 μL min^{-1}. The LC gradient starts with 95% solvent A and 5% solvent B, and this ratio was kept until 1.00 min, then increased solvent B to 100% until 12.00 min, and kept the ratio until 13.00 min. The mass spectrometer TSQ Quantiva (Thermo Fisher Scientific, San Jose, CA) was operated with a high temperature ESI source in negative mode. The ion source related parameters were: spray voltage: static; negative ion: 3200 V; sheath gas: 38.3 Arb; aux gas: 1.2 Arb; sweep gas: 2.8 Arb; Ion transfer tube temp: 325 °C; vaporizer temp: 50 °C; CID gas: 1.5 mTorr. The PFAS stock solutions were prepared in methanol to a final concentration of 1 mg mL^{-1} and stored at 4 °C. The calibration solutions were diluted with water to the corresponding concentration, and all calibration solutions and samples contain the internal standards with a spiked concentration of 5 μg/L.

Supplementary note 6: Life-cycle assessment. A cradle-to-gate, multi-dimensional, life cycle assessment (LCA) was carried out to quantify the environmental impacts of RAPIMER production and use. We examined environmental impacts acidification, greenhouse gas emissions, human toxicity (cancer and non-cancer), ecotoxicity, ozone depletion, particulate matter, and surface ozone formation. The analysis was done using OpenLCA 1.10.3 software (https://www.openlca.org/) over data primarily adopted from the Ecoinvent 3.7 database. For comparative purposes, we examined the RAPIMER results with that from two commonly proposed PFAS sorbents, namely activated carbon and ion exchange resins.

We performed two sets of comparisons. First, we directly compared the environmental impacts for producing one kg of the respective sorbent. Second, we normalized the comparison by examining the environmental impacts in treating 1 m^3 of PFAS contaminated groundwater (with a PFAS concentration rate of 0.21 μg/L) using each of the three sorbents [1]. Particularly, we factored in sorbents' different adsorption capacities in doing the normalization. For instance, the equilibrium adsorption capacity of the RAIPMER ranges between 3.53 to 4.15 kg PFAS/kg sorbent for PFAS (PFOA and PFOS), which is much higher than that of activated carbon at 0.4-0.42 kg/kg[2,3] and anion exchange resin at 1.5-3.07 kg/kg[4,5], respectively.

Mathematically, the calculation takes the form as follows.

$$NEI_{we} = EI_{we} \times \frac{c}{cap_w} \qquad (4)$$

Where w indicates the sorbent form (RAPIMER, activated carbon, or ion exchange resins) and e the form of environmental impact (GHGs, ozone etc). EI_{we} refers to the environmental impacts of type e arising when producing one kg of sorbent w as we discuss further below. c is the total PFAS contained in the polluted groundwater; that is 0.21 μg/L×1000 L = 0.00021 kg. cap_w denotes the sorbent equilibrium adsorption capacities in terms of kg PFAS absorbed per kg of sorbent. For that we used the mean of its absorbent capacity range (i.e., 3.84 kg PFAS per kg of RAIPMER, 0.41 kg PFAS per kg of activated carbon, and 2.28 kg PFAS per kg of anion exchanged resins) [2,3,4,5]. Finally, NEI_{we} is the resultant computation for environmental impact of type e for the inputs used in making the sorbent for treating 1 m^3 of PFAS contaminated groundwater. For RAPIMER we took the amounts of each of the chemicals and other inputs used to make 1 kg of sorbent using the items listed in the methods section. We then looked up their multi-dimensional impacts (e) from the Ecoinvent 3.7 database then added across all inputs to get the multi-dimensional environmental impact vector per kg of RAPIMER. For activated carbon and anion exchanged resins we drew the impact vector per kg directly from the Ecoinvent database 3.7.1[6]. For computing emissions related to energy use, we assume the electricity employed was clean and renewable (i.e., hydrological power), which is line with the "net-zero" emission initiatives to combat climate change[2] (Table S5).

Supplementary Figures

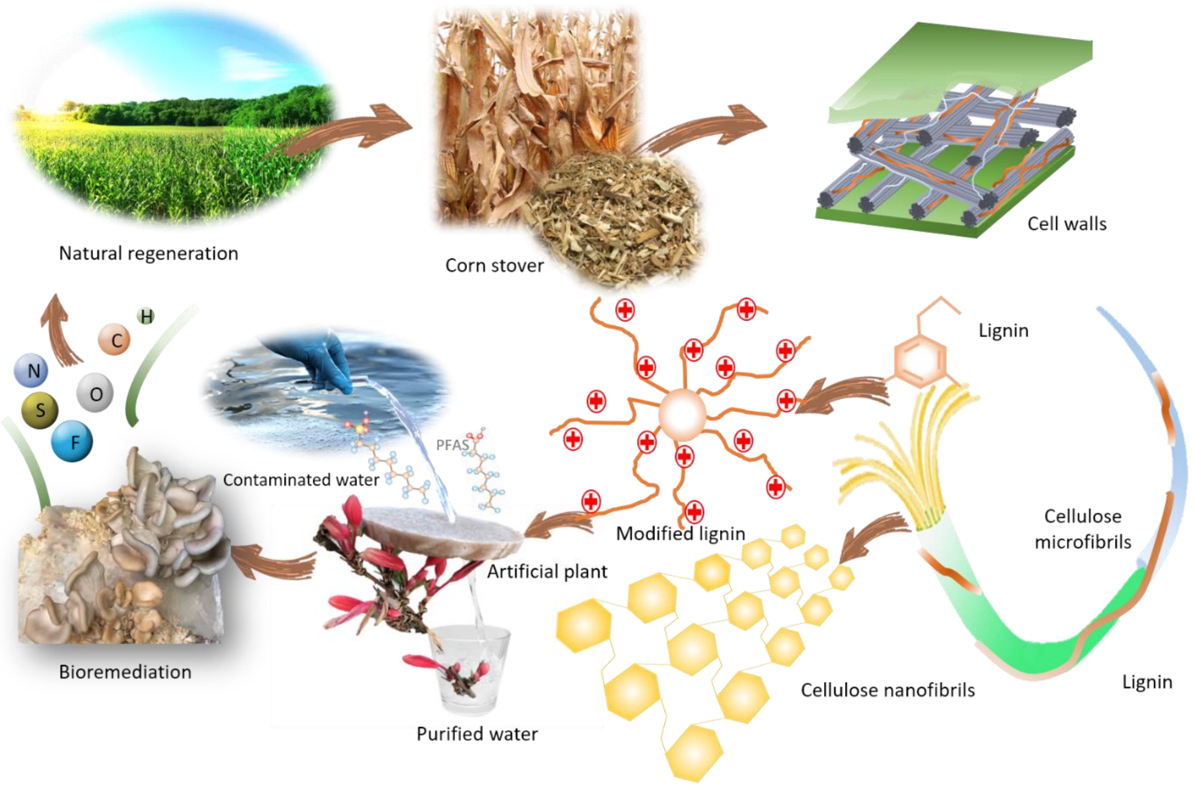

Supplementary Figure 1. The concept and mechanism of the RAPIMER system for PFAS removal and biodegradation.

Both constituent materials, cellulose and lignin, were produced from corn stover residue and then reverse-engineered to develop the RAPIMER composite. The PFAS enriched RAPIMER composite worked like plant cell wall as the sole carbon source to sustain fungus growth and was synergistically biodegraded.

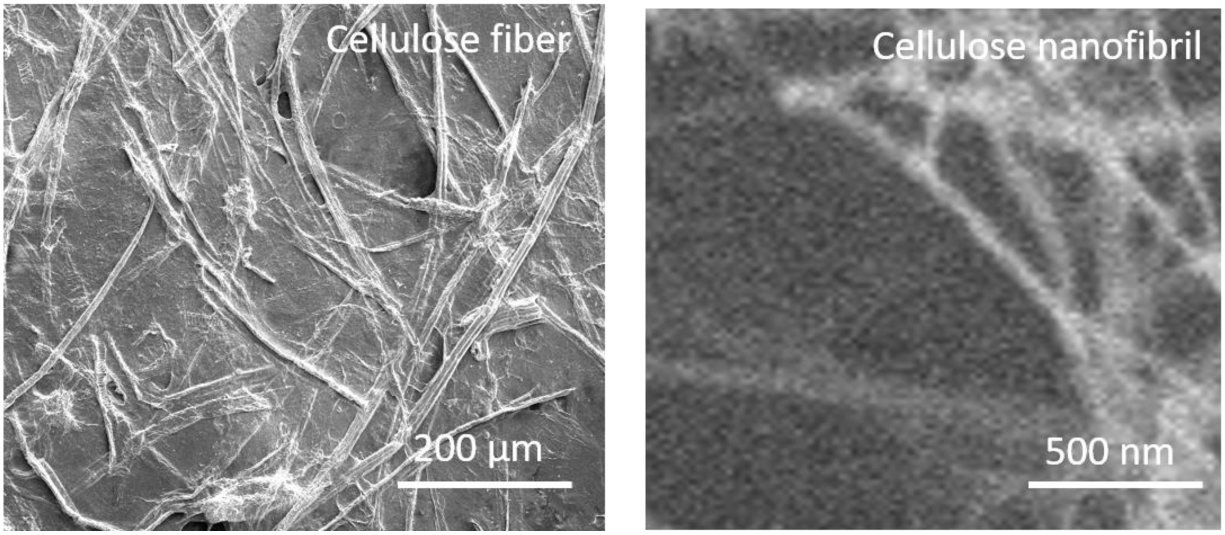

Supplementary Figure 2. The SEM images of cellulose fibers and cellulose nanofibrils. The morphologies of cellulose fibers and cellulose nanofibrils showed the fiber dimeters of cellulose nanofibrils were significantly reduced from cellulose fibers after TEMPO-oxidation process. The experiment was reproduced n=3 times.

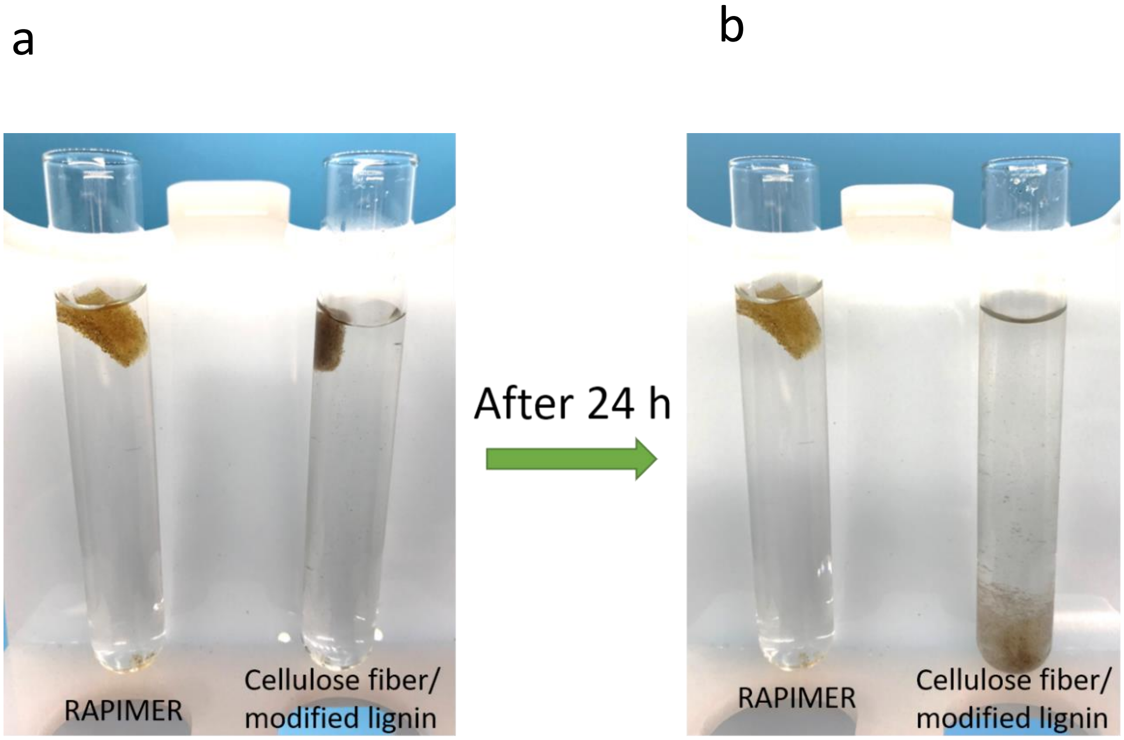

Supplementary Figure 3. Hydrostability of different material composites.

Supplementary Figure 3 a showed that the RAPIMER and cellulose fiber/modified lignin composites were immersed in the DI water. Supplementary Figure 3b showed that the RAPIMER composites were immersed in the DI water after 24 hours. The RAPIMER composite maintained its structure while the cellulose fiber/modified lignin composite separated in the water after 24 hours.

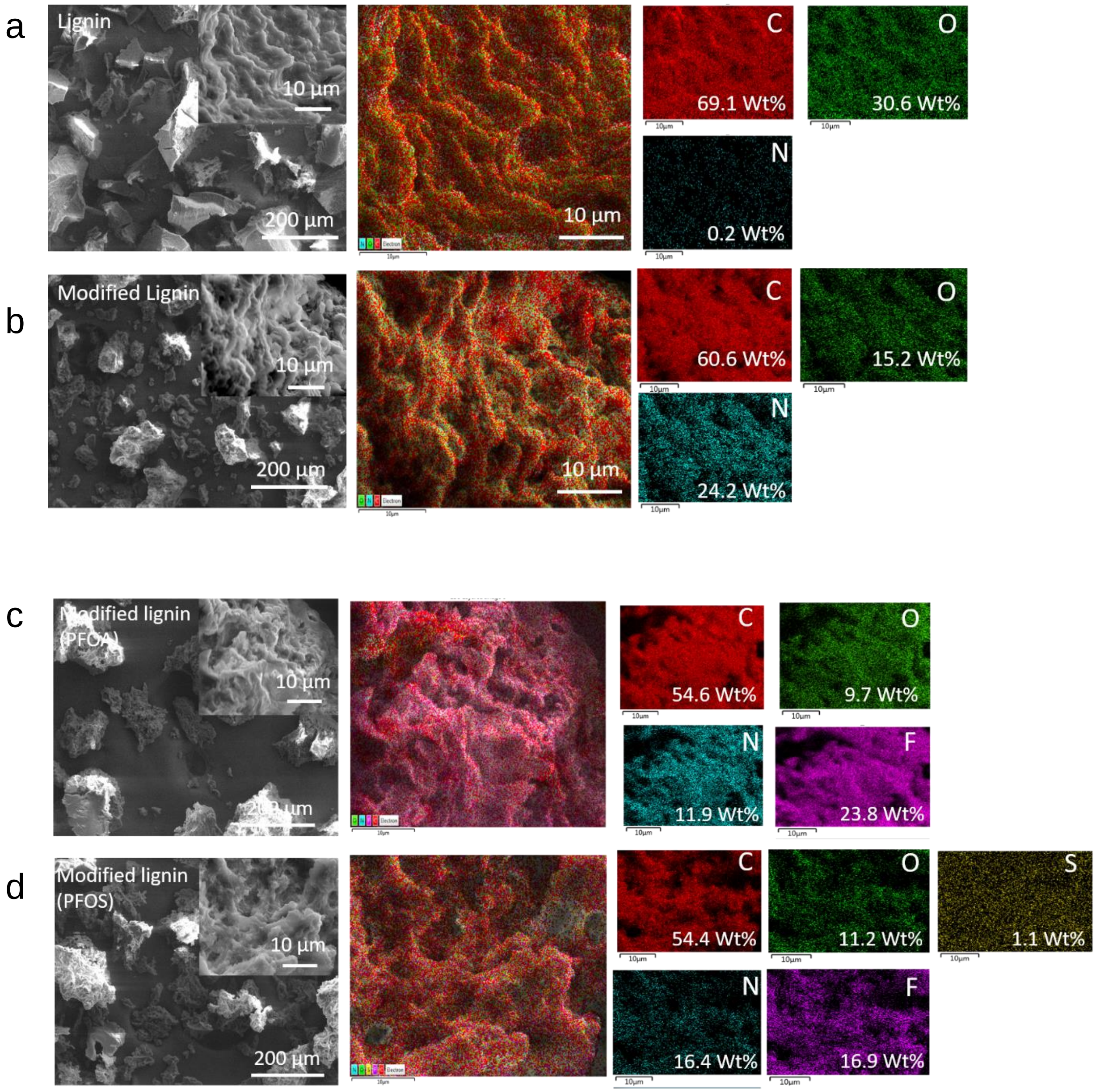

Supplementary Figure 4. EDX images of lignin and modified lignin.

Fig S4 a and b showed the morphologies of lignin and modified lignin with element analysis, respectively. The result demonstrated that the content of nitrogen element significantly increased in modified lignin compared with lignin, which indicated the polyethyleneimine was successfully grafted on lignin. Supplementary Figure 4 c and d showed the morphologies of modified lignin with element analysis after adsorbing PFOA and PFOS, respectively. The results indicated that both PFOA and PFOS were adsorbed by the modified lignin, in which the fluoride and sulfur elements were detected by EDX.

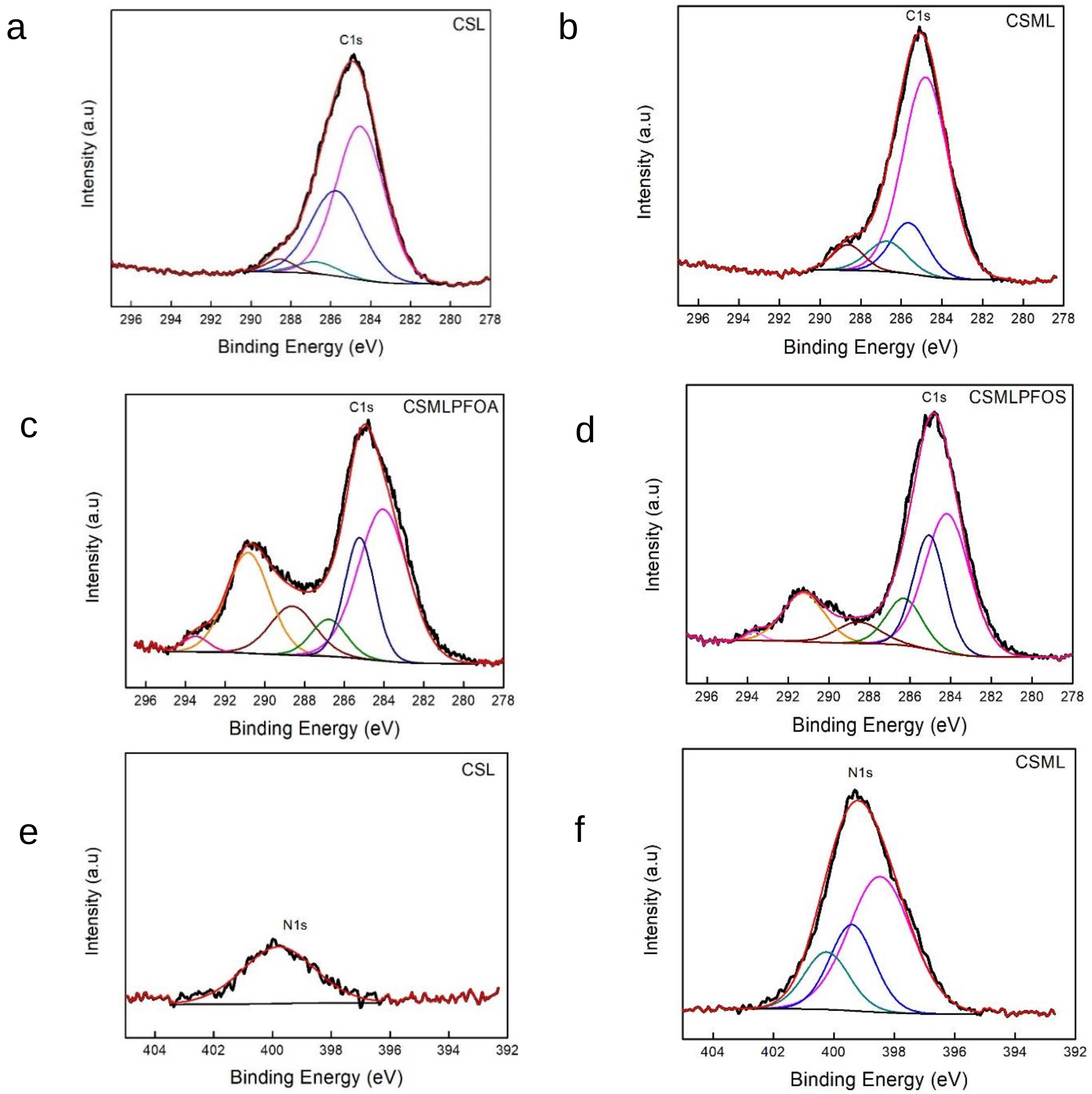

Supplementary Figure 5. The details of XPS image (Fig. 2k) in carbon peak and nitrogen peak of lignin and modified lignin before and after PFAS adsorption.

Supplementary Figure 5 a and b are carbon peaks for lignin and modified lignin, respectively. The lignin and modified lignin spectra indicated the presence of four types of carbon atoms in different functional groups: The hydrocarbon (C=C/C-C 284.5 eV), noncarbonylic carbon (C-O, 285.8 eV), carbonyl carbon (C=O 286.7 eV), and carboxylate carbon (O=C-O 288.6 eV). The peak intensity and ratio of C=C/C-C carbon in the modified lignin significantly increased compared to that of lignin, which indicated that the polyethyleneimine with C=C/C-C carbon was effectively grafted on the lignin during the chemical

reaction. Fig S5 c and d are carbon peaks for modified lignin after adsorbing PFOA and PFOS. Compared to the spectra of lignin and modified lignin (Supplementary Figure 5 a and b), the high-resolution XPS C1s spectra of the modified lignin after adsorbing PFOA and PFOS showed three more peaks representing (C-F 288.5 eV), ($-CF_2$ 291.7 eV), and ($-CF_3$ 294.0 eV), respectively. It indicated that the PFOA and PFOS have been adsorbed and immobilized on the surface of the modified lignin. Fig S5 e and f are the nitrogen peaks of lignin and modified lignin. The high-resolution XPS N1s spectra of the lignin and modified lignin showed the intensity significantly increased in modified lignin compared with that of lignin. The three peaks could be fitted at 398.1 eV, 398.7 eV, and 399.3 eV, which could be considered as secondary amino, primary amino, and tertiary amino, respectively. This indicated that the amino groups on polyethyleneimine had grafted onto the lignin as a cationic ion in the RAPIMER systems. The XPS data generated in this study are provided in the Source Data file.

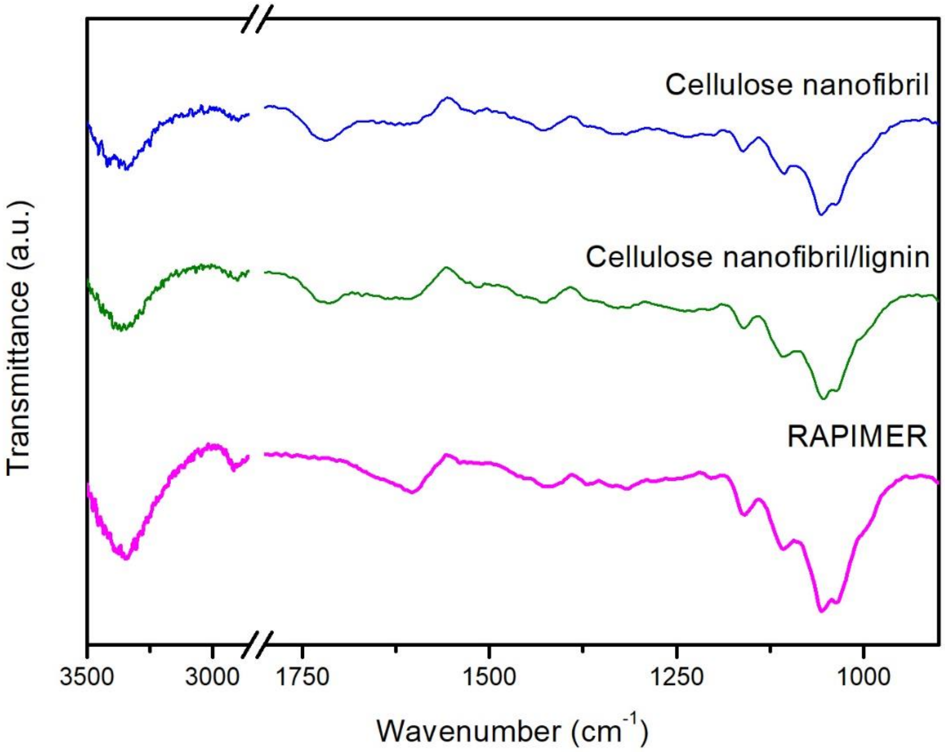

Supplementary Figure 6. FTIR spectra of cellulose nanofibril, cellulose nanofibril/lignin composite, and the RAPIMER composite. The peak of free carboxyl groups (1720 cm^{-1} in both blue cellulose nanofibril composite and cellulose nanofibril/lignin composite spectra) was entirely shifted to that of carboxylate groups (1600 cm^{-1} in the purple RAPIMER spectrum) by the formation of carboxylic acid/amine salt. The FTIR data generated in this study are provided in the Source Data file.

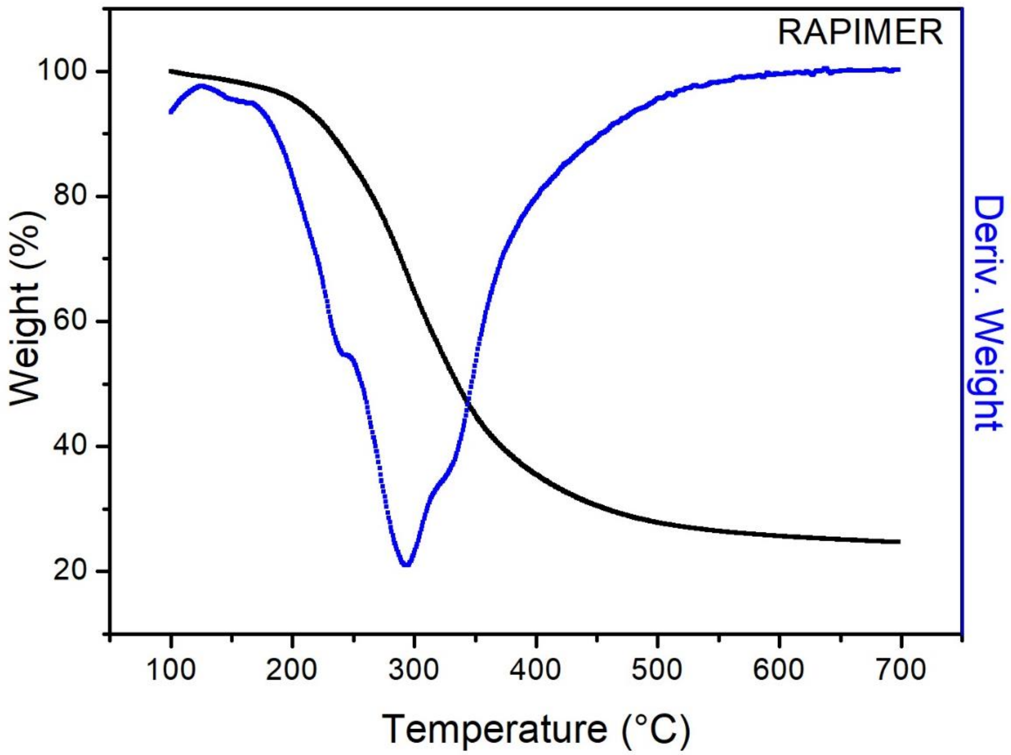

Supplementary Figure 7. TGA analysis of the RAPIMER composite. Black solid line, percentage weight loss of the RAPIMER composite. Blue solid line, derivative weight of the composite material. The RAPIMER composite thermal degradation started around 200 °C. The TGA data generated in this study are provided in the Source Data file.

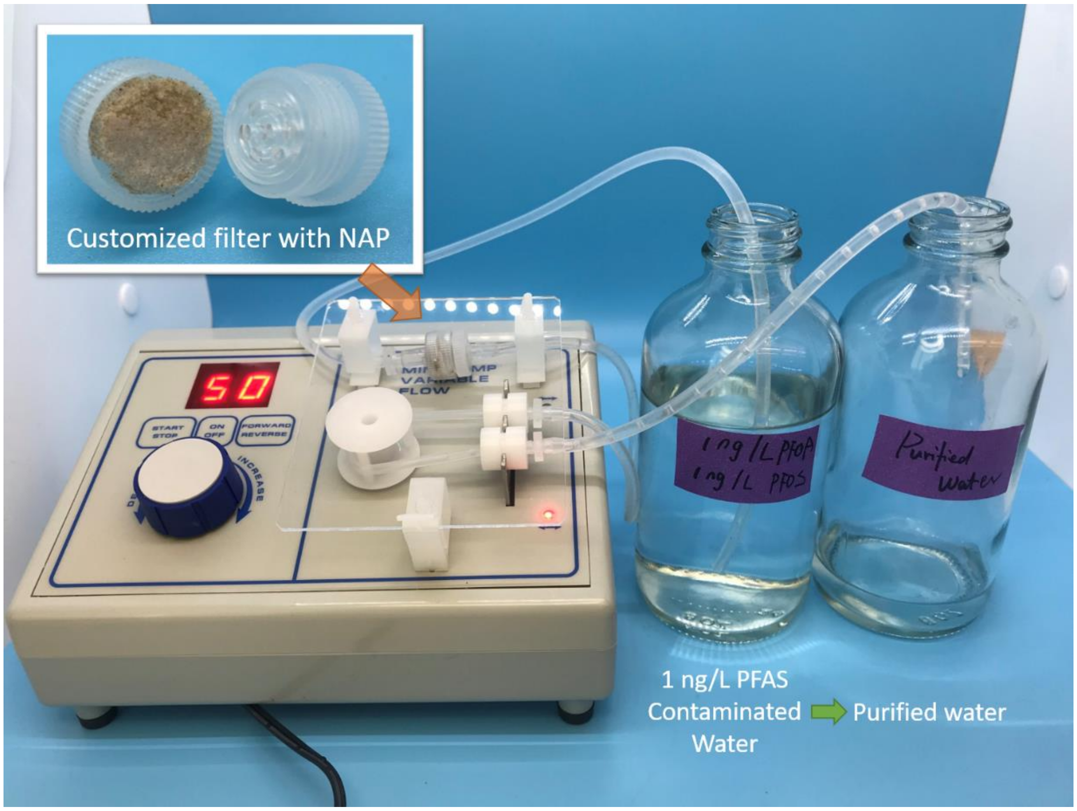

Supplementary Figure 8. Customized filter packed with RAPIMER composite for 1 µg/L and 10 µg/L PFOA and PFOS adsorption testing.

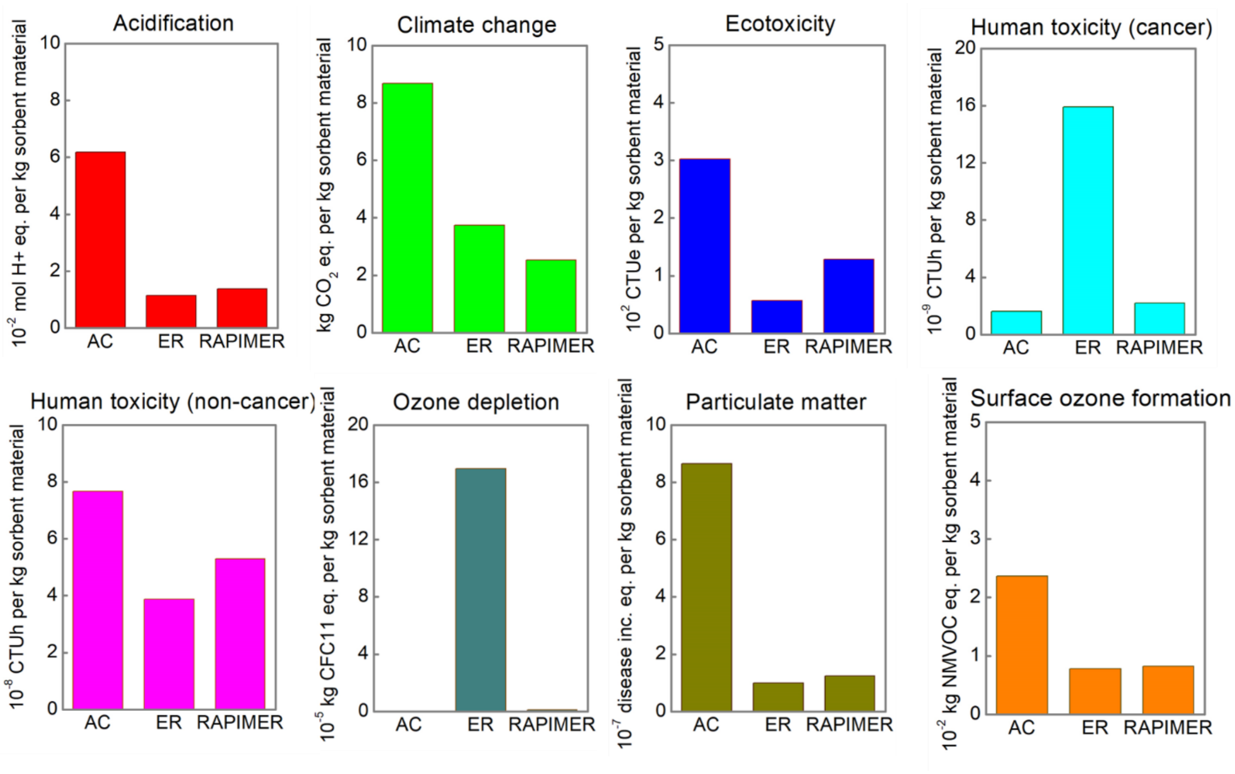

Supplementary Figure 9. The life cycle impact assessment results per kg of the activated carbon (AC), exchanged resin (ER) and RAPIMER. CTUe, comparative toxicity unit for ecotoxicity, CTUh, comparative toxicity unit for human health; CFC-11, (trichlorofluoromethane) equivalent; NMVOC, non-methane volatile organic compound. The LCA data generated in this study are provided in the Source Data file.

Supplementary Tables.

Table S1. Density, porosity, and specific surface area of cellulose materials

Cellulose	Bulk density (kg/m^3)	Structural porosity (%)	Specific surface area(m^2/g)
Cellulose fibers	15.59	99.03%	13.10
Cellulose nanofibrils	8.65	99.40%	177.96

Table S2. Calculated constants of the pseudo-second-order model of PFOA and PFOS on the RAPIMER system, sorbents, and controls

Sorbents	adsorbates	The pseudo-second-order model		
		q_e (mg/g)	k	R^2
Cellulose fiber/ modified lignin	PFOA	2410	2.057×10^{-5}	0.977
RAPIMER	PFOA	3529	5.951×10^{-5}	0.986
Modified lignin	PFOA	4104	4.355×10^{-5}	0.992
Cellulose fiber/ modified lignin	PFOS	3658	1.051×10^{-5}	0.936
RAPIMER	PFOS	4151	1.703×10^{-5}	0.989
Modified lignin	PFOS	4262	1.210×10^{-4}	0.979
Control (cellulose fiber/lignin)	PFOA	3.9	-0.698	0.193
Control (cellulose nanofibril/lignin)	PFOA	19.4	-0.1208	0.187
Control (cellulose fiber/lignin)	PFOS	225	3028	0.150
Control (cellulose nanofibril/lignin)	PFOS	128.1	-689	0.0175

Table S3. Comparison of studies on different sorbents for PFOA and PFOS removal from water

Sorbent	Target PFAS	Initial PFAS Concentration (mg/L)	Adsorption Capacity (Q_{max} mg/g)	Time to maximum adsorption n (h)	PH	Reference
Modified lignin	PFOA, PFOS	0.000001-400	PFOA: 4104<br>PFOS: 4262	5-10	2-11	Our work
RAPIMER	PFOA, PFOS	0.000001-400	PFOA: 3529<br>PFOS: 3203	24-48	2-11	Our work
Aminated rice husk	PFOA, PFOS	10-15	PFOA:1030<br>PFOS: 1325	3-6	4-11	[7]
MWCNTs-electrode	PFOA, PFOS	0.05-10	PFOA: 405.8<br>PFOS: 505.7	3	6.5	[8]
Oxidized MWCNTs	PFOA, PFOS	0.05-50	PFOA: 132.5<br>PFOS: 381.9	10	5	[9]
CNTs-20% graphene	PFOA, PFOS	0.1-10	PFOA: 491.9<br>PFOS: 555.8	10	5	[10]
Activated carbon	PFOA, PFOS	1-200	PFOA: 476.2<br>PFOS: 1160.3	24	3-10	[11]

Hexagonal mesoporous silica (HMS)	PFOS, PFOS	25-30	PFOA: 70<br>PFOS: 19	1-3	7	[12]
Magnetic mesoporous carbon nitride	PFOA, PFOS	80-90	PFOA: 370<br>PFOS: 455	0.5-1	2-9	[13,14]
Resin [Amb IRA-400]	PFOA, PFOS	5	PFOA: 1200<br>PFOS: 200	120-200	3-7	[15]
Amine-grafted Metal-Organic-Frameworks (MOFs)	PFOA	50-300	PFOA 290-750	1-2	3-10	[16]
β-Cyclodextrin Copolymer	PFOA	400-8000	PFOA: 300-400	2-5	3.8	[17]
Quaternized Cotton	PFOA, PFOS	50--500	PFOA:1240<br>PFOS: 1750	3-9	3-10	[18]
Chitosan-based molecularly imprinted polymer (MIP)	PFOS	50	PFOS: 1460	30-50	3	[19]

Table S4. Calculated constants of the Langmuir and Freundlich equations for the adsorption of PFOA and PFOS on the modified lignin particles and the RAPIMER composite

Sorbents	adsorbates	Langmuir model			Freundlich model		
		q_m (mg/g)	b (mg/L)	R^2	K_f	n	R^2
Modified lignin	PFOA	18529.6	0.0036	0.985	253.9	1.58	0.958
RAPIMER	PFOA	3203.0	0.0140	0.925	354.6	2.87	0.842
Modified lignin	PFOS	11876.8	0.0061	0.984	380.3	1.90	0.941
RAPIMER	PFOS	3000.0	0.0087	0.876	163.6	2.20	0.890

Table S5. Information on the input material to produce 1 kg of the RAPIMER

Input Material	Quantity	Unit	Inventory data sources
**Agricultural residue**			
Corn stover	2.5	kg	USLCI
**Chemical input**			
Sodium hydroxide	275	g	Ecoinvent 3.7
Polyethyleneimine (PEI)	160	g	Ecoinvent 3.7
Formaldehyde	593	g	Ecoinvent 3.7
Sodium hypochlorite	438	g	Ecoinvent 3.7
Citric acid	50	g	Ecoinvent 3.7
**Energy input**			
Electricity [a]	27	kWh	Ecoinvent 3.7
**Output**			
RAPIMER	1	kg	

[a] The electricity consumption during the production of RAPIMER was considered to use industrial processes (i.e. freeze-drying, and etc.). In our process, we assume the power required for drying 1 kg RAPIMER is 2.20 kWh based on the literature and actual electrical comsumption[20]. The rest of electricity usage was estimated based on the watts of the equipment and the conditioning time.

Table S6. The maximum adsorption capacity of typical Sorbents and RAPIMER for PFAS treatment

Sorbent	Isothermal maximum adsorption capacity(mg/g)	Ref.
Activated carbon	400-415	[2,3]
Exchanged resin (IRA67)	1500-3067	[4,5]
RAPIER	3529-4151	This work

References

1 Guelfo, J. L. & Adamson, D. T. Evaluation of a national data set for insights into sources, composition, and concentrations of per-and polyfluoroalkyl substances (PFASs) in US drinking water. *Environmental Pollution* **236**, 505-513 (2018).

2 Du, Z. *et al.* Removal of perfluorinated carboxylates from washing wastewater of perfluorooctanesulfonyl fluoride using activated carbons and resins. *Journal of hazardous materials* **286**, 136-143 (2015).

3 Zhang, Q., Deng, S., Yu, G. & Huang, J. Removal of perfluorooctane sulfonate from aqueous solution by crosslinked chitosan beads: sorption kinetics and uptake mechanism. *Bioresource technology* **102**, 2265-2271 (2011).

4 Yu, J., Hu, J., Tanaka, S. & Fujii, S. Perfluorooctane sulfonate (PFOS) and perfluorooctanoic acid (PFOA) in sewage treatment plants. *Water research* **43**, 2399-2408 (2009).

5 Deng, S., Yu, Q., Huang, J. & Yu, G. Removal of perfluorooctane sulfonate from wastewater by anion exchange resins: Effects of resin properties and solution chemistry. *Water Research* **44**, 5188-5195 (2010).

6 Moreno Ruiz, E. *et al.* Documentation of changes implemented in ecoinvent database v3. 7 & v3. 7.1. ecoinvent Association. *Zürich, Switzerland* (2020).

7 Deng, S. *et al.* Adsorption of perfluorinated compounds on aminated rice husk prepared by atom transfer radical polymerization. *Chemosphere* **91**, 124-130 (2013).

8 Li, M., Yu, Z., Liu, Q., Sun, L. & Huang, W. Photocatalytic decomposition of perfluorooctanoic acid by noble metallic nanoparticles modified TiO2. *Chemical Engineering Journal* **286**, 232-238 (2016).

9 Li, X. *et al.* Adsorption of ionizable organic contaminants on multi-walled carbon nanotubes with different oxygen contents. *Journal of hazardous materials* **186**, 407-415 (2011).

10 Niu, Z. *et al.* Electrochemically enhanced removal of perfluorinated compounds (PFCs) from aqueous solution by CNTs-graphene composite electrode. *Chemical Engineering Journal* **328**, 228-235 (2017).

11 Deng, S. *et al.* Enhanced adsorption of perfluorooctane sulfonate and perfluorooctanoate by bamboo-derived granular activated carbon. *Journal of hazardous materials* **282**, 150-157 (2015).

12 Punyapalakul, P., Suksomboon, K., Prarat, P. & Khaodhiar, S. Effects of surface functional groups and porous structures on adsorption and recovery of perfluorinated compounds by inorganic porous silicas. *Separation Science and Technology* **48**, 775-788 (2013).

13 Yan, T., Chen, H., Jiang, F. & Wang, X. Adsorption of perfluorooctane sulfonate and perfluorooctanoic acid on magnetic mesoporous carbon nitride. *Journal of Chemical & Engineering Data* **59**, 508-515 (2014).

14 Yan, T., Chen, H., Wang, X. & Jiang, F. Adsorption of perfluorooctane sulfonate (PFOS) on mesoporous carbon nitride. *RSC advances* **3**, 22480-22489 (2013).

15 Yu, Q., Zhang, R., Deng, S., Huang, J. & Yu, G. Sorption of perfluorooctane sulfonate and perfluorooctanoate on activated carbons and resin: kinetic and isotherm study. *Water research* **43**, 1150-1158 (2009).

16 Liu, K. *et al.* Understanding the adsorption of PFOA on MIL-101 (Cr)-based anionic-exchange metal–organic frameworks: comparing DFT calculations with aqueous sorption experiments. *Environmental science & technology* **49**, 8657-8665 (2015).

17 Karoyo, A. H. & Wilson, L. D. Tunable macromolecular-based materials for the adsorption of perfluorooctanoic and octanoic acid anions. *Journal of colloid and interface science* **402**, 196-203 (2013).

18 Deng, S. *et al.* Highly efficient sorption of perfluorooctane sulfonate and perfluorooctanoate on a quaternized cotton prepared by atom transfer radical polymerization. *Chemical engineering journal* **193**, 154-160 (2012).

19 Yu, Q., Deng, S. & Yu, G. Selective removal of perfluorooctane sulfonate from aqueous solution using chitosan-based molecularly imprinted polymer adsorbents. *Water Research* **42**, 3089-3097 (2008).
20 Prosapio, V., Norton, I. & De Marco, I. Optimization of freeze-drying using a Life Cycle Assessment approach. (2017).
